# Altered hippocampal functional connectivity after the rupture of anterior communicating artery aneurysm

**DOI:** 10.3389/fnagi.2022.997231

**Published:** 2022-11-07

**Authors:** Fuxiang Chen, Jiawei Cai, Linsun Dai, Yuanxiang Lin, Lianghong Yu, Zhangya Lin, Yaqing Kang, Ting Yu, Dengliang Wang, Dezhi Kang

**Affiliations:** ^1^Department of Neurosurgery, Neurosurgery Research Institute, The First Affiliated Hospital, Fujian Medical University, Fuzhou, China; ^2^Department of Neurosurgery, National Regional Medical Center, Binhai Campus of the First Affiliated Hospital, Fujian Medical University, Fuzhou, China; ^3^Fujian Provincial Institutes of Brain Disorders and Brain Sciences, First Affiliated Hospital, Fujian Medical University, Fuzhou, China; ^4^Department of Radiology, The First Affiliated Hospital of Fujian Medical University, Fuzhou, China

**Keywords:** anterior communicating artery aneurysm, subarachnoid hemorrhage, hippocampus, cognitive impairment, functional connectivity, Papez circuit

## Abstract

**Background and purpose:**

Aneurysmal subarachnoid hemorrhage (SAH) predisposes hippocampal injury, a major cause of follow-up cognitive impairment. Our previous study has revealed an abnormal resting-state brain network in patients after the rupture of anterior communicating artery (ACoA) aneurysm. However, the functional connectivity (FC) characteristics of the hippocampus and its relationship with cognitive performance in these patients remain unknown.

**Methods:**

This study ultimately included 26 patients and 19 age- and sex-matched controls who completed quality control for resting-state functional magnetic resonance imaging (fMRI). The mean time series for each side of the hippocampus was extracted from individuals and then a seed-to-voxel analysis was performed. We compared the difference in FC strength between the two groups and subsequently analyzed the correlations between abnormal FC and their cognitive performance.

**Results:**

The results of bilateral hippocampus-based FC analysis were largely consistent. Compared with the healthy controls, patients after the rupture of ACoA aneurysm exhibited significantly decreased FC between the hippocampus and other brain structures within the Papez circuit, including bilateral anterior and middle cingulate cortex (MCC), bilateral medial superior frontal gyrus, and left inferior temporal gyrus (ITG). Instead, increased FC between the hippocampus and bilateral insula was observed. Correlation analyses showed that more subjective memory complaints or lower total cognitive scores were associated with decreased connectivity in the hippocampus and several brain regions such as left anterior cingulate cortex (ACC) and frontotemporal cortex.

**Conclusion:**

These results extend our previous findings and suggest that patients with ruptured ACoA aneurysm exist hypoconnectivity between the hippocampus and multiple brain regions within the Papez circuit. Deactivation of the Papez circuit may be a crucial neural mechanism related to cognitive deficits in patients after the rupture of ACoA aneurysm.

## Introduction

Subarachnoid hemorrhage (SAH) caused by the rupture of anterior communicating artery (ACoA) aneurysm is a devastating cerebrovascular event with high morbidity and mortality (Hong et al., 2012; Ito et al., 2015). Cognitive impairment reportedly occurs in approximately half of the surviving patients, of which memory loss is the most common complaint, preventing them from returning to their normal life (Beeckmans et al., 2020; Neifert et al., 2021). Therefore, there is an urgent clinical need for the treatment of cognitive dysfunction following the rupture of cerebral aneurysm. However, the neural mechanisms underlying cognitive deficits after the rupture of ACoA aneurysm remain unclear, resulting in limited treatment.

Numerous neuroimaging studies have been performed to explore cognitive deficits caused by neurological problems, including hemorrhagic cerebrovascular disease (Baldassarre et al., 2016; Siegel et al., 2016; Adhikari et al., 2017; Chung et al., 2021). There was compelling evidence that cognitive dysfunction is associated with an aberrant functional brain network (Maher et al., 2015; Mikell et al., 2015; Nelson et al., 2016). It is well established that resting-state functional magnetic resonance imaging (fMRI) reflects the spontaneous neural activity of the brain and is of great value for assessing brain network characteristics (Fox et al., 2014). Unexpectedly, few studies concentrated the effect of ruptured cerebral aneurysms on brain activity and intrinsic connectivity (Maher et al., 2015; Mikell et al., 2015; Su et al., 2018). A domestic study using resting-state fMRI revealed significant abnormal functional connectivity (FC) of medial temporal lobe and thalamus in the group of patients with SAH as compared to the healthy controls (Su et al., 2018). Furthermore, the decreased FC between several brain regions, such as left thalamus-left inferior parietal lobe and left inferior temporal gyrus (ITG)-bilateral inferior frontal gyrus, was significantly associated with their poor memory performance (Su et al., 2018). Another two resting-state fMRI studies focused primarily on post-SAH executive function impairment, in which disruption of frontal networks or frontoparietal connectivity in the patients' group was found (Maher et al., 2015; Mikell et al., 2015). These studies to a certain extent shed light on the functional brain network mechanisms of cognitive dysfunction after the rupture of cerebral aneurysm.

Notably, the location of ruptured aneurysms in the abovementioned three studies was not unique that would undoubtedly lead to different patterns of structural brain damage, and further had a non-negligible impact on the findings of functional brain network analysis. Take the example of ruptured anterior circulation aneurysms, damage to adjacent brain structures and the probability of vasospasm are both related to their locations. Therefore, we found that patients suffering from the rupture of ACoA aneurysm clinically tend to present more severe symptoms (Beeckmans et al., 2020; Neifert et al., 2021). In addition, hydrocephalus is a common sequela after aneurysmal SAH that usually leads to cognitive deficits. Similarly, epileptic seizure secondary to the rupture of cerebral aneurysm has also been reported to induce long-term cognitive function decline (Taufique et al., 2016). Hence, these confounding factors should be considered together to better understand the intrinsic network mechanisms of cognitive dysfunction after aneurysmal SAH and to accurately provide characteristic brain network markers for that trouble.

As the ACoA aneurysms locate in the anterior interhemispheric region, the medial prefrontal cortex (mPFC) is theoretically more susceptible to mechanical insult due to its close distance in anatomy when an aneurysm ruptures. The medial superior frontal gyrus (SFGmed) is a principal component of mPFC, which is involved in a variety of cognitive processes (Rolls et al., 2022). Therefore, we recently set the SFGmed as the region of interest (ROI) to perform a seed-based analysis and found significantly decreased resting-state FC of the SFGmed in patients with ruptured ACoA aneurysm. Notably, resting-state images of these patients were acquired after surgical clipping or endovascular coiling. Although the quality control of these imaging data was accomplished according to standard procedures, we should not completely ignore the effect of metal artifacts on fMRI data analysis (Khursheed et al., 2011). Furthermore, the SFGmed consists of presupplementary motor area and supplementary motor area, which play an important role in cognitive control and motor, respectively, indicating different roles of subregions (Zhang et al., 2012). Therefore, another brain region involvement of cognitive function, simultaneously distant from the aneurysm, and capable of functionally similar anatomical subregions may be more preferred.

The hippocampus is generally considered one of the brain regions most affected by aneurysmal SAH and is closely correlated with follow-up cognitive function decline, especially memory loss (Wostrack et al., 2014; Veldeman et al., 2017). Accumulating evidence from the SAH model of animal and neuroimaging studies has revealed that patients following the rupture of cerebral aneurysms present structural and functional changes in the hippocampus (Bendel et al., 2009; Sherchan et al., 2011; Wostrack et al., 2014), including structural atrophy, white matter degeneration, and hypoactivity (Wostrack et al., 2014; Cho and Jang, 2020). In our previous study, decreased FC between the hippocampus and the SFGmed was found in patients after the rupture of ACoA aneurysm, which was also extensively demonstrated in abundant studies concerning cognitive function. In addition, published studies have revealed that hippocampal activity was increased after running training or non-invasion brain stimulation (Huiskamp et al., 2020; Niu et al., 2022), suggesting an ability of neuroplasticity. In addition, neuromodulation targeting directly the hippocampus was considered a potential and promising alternative method to some non-acoustic brain stimulation modalities (Huang et al., 2022). Taken together, investigating the hippocampal resting-state FC in patients with a history of ACoA aneurysm rupture is reasonable and meaningful.

Therefore, this study is an extension of our previous study on functional brain network in patients after the rupture of ACoA aneurysm. The aim of this study is to explore the alterations of bilateral hippocampal connectivity in patients with ruptured ACoA aneurysm and their relationship with impaired cognitive function. We hypothesized the decline in hippocampal connectivity in patients with SAH due to ruptured ACoA aneurysm as compared to healthy controls, and abnormalities of some network features were related to their cognitive performance.

## Materials and methods

### Participants

We recruited patients with a history of ACoA aneurysm rupture for more than 6 months from the database of the First Affiliated Hospital of Fujian Medical University. Other inclusion criteria were age ranging from 35 to 70 years old and normal cognitive function before the SAH onset. Exclusion criteria were (1) neuropsychiatric diseases or previous stroke; (2) occurrence of epileptic seizure or delayed cerebral ischemia during hospitalization; and 3) hydrocephalus secondary to SAH. The original participants consisted of 27 (16 males, mean age 56.5 years, range 36–74) patients and 20 (11 males, mean age 53.3 years, range 49–68) age- and sex-matched healthy controls. All participants were right-handed. The local Ethics Committee approved this study, and all subjects gave their written informed consent.

### MRI acquisition

All structural imaging and resting-state fMRI scanning were obtained by the same 3.0-T imaging scanner (Siemens Medical Solutions, Germany). T1-weighted structural images were acquired with the following parameters: repetition time (TR) = 2,300 ms, echo time (TE) = 3.09 ms, field of view (FOV) = 256 × 240 mm, flip angle = 9°, matrix = 256 × 256, voxel size = 1.0 × 1.0 × 1.0 mm^3^, 192 sagittal slices, thickness = 1 mm, and spaced = 0.5 mm. Earplugs and foam padding were used to minimize scanner noise and restrict head motion. When the resting-state fMRI acquisition starts, individuals were asked to stay awake and to think of nothing. Resting-state data were collected with the following parameters: TE = 30 ms, TR = 3,000 ms, flip angle = 90°, FOV = 240 × 240 mm, matrix = 80 × 80, voxel size = 3.0 × 3.0 × 3.4 mm^3^, 50 slices with no gap, and thickness = 3.4 mm. A total of 205 time points was collected for individuals, and the fMRI data were acquired over 10 min. All participants were inspected by a radiologist to exclude morphological abnormalities.

### Assessment of cognitive function

Cognitive function assessment was carried out after fMRI acquisition by two researchers who were blinded to this study. According to a previous report (Kim et al., 2021), the Subjective Memory Complaints Questionnaire (SMCQ) and the Montreal Cognitive Assessment (MoCA) were used to measure subjective memory problems and cognitive status, respectively.

### Data preprocessing

Resting-state data preprocessing was performed by the Data Processing Assistant for Resting-State fMRI (DPARSF; http://restfmri.net/forum/DPARSF). The first 10 time points of fMRI data were discarded from the analysis in consideration of magnetization stabilization and the adaption of subjects to the scanning environment. Standard preprocessing steps were performed as previously reported, including the following: (1) slice timing (reference slice was the middle slice); (2) head motion correction; (3) spatially normalized to the Montreal Neurological Institute template (voxel size was resampled to 2 mm isotropic voxels); (4) smoothing with a Gaussian filter by 8 mm full width at half-maximum; (5) linear detrending; (6) bandpass temporal filtering (0.01–0.08 Hz); and (7) regressing out nuisance covariates (Friston 24 head motion parameters, whiter matter signal, and cerebrospinal fluid signal).

**Figure 1:**
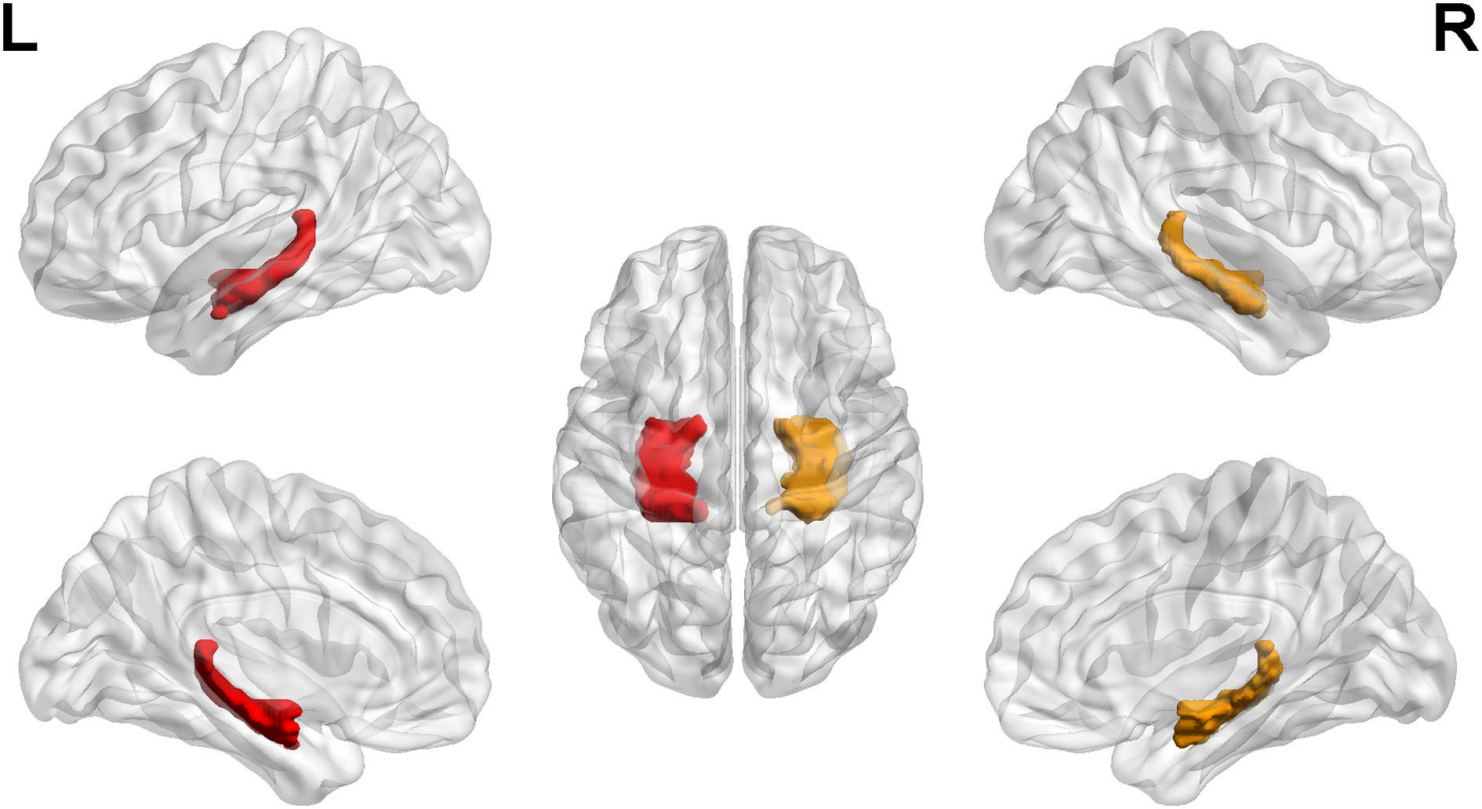
Regions of interest. The left hippocampus and right hippocampus labeling with color. L, left; R, right.

Then, binary masks of the left hippocampus and the right hippocampus were chosen to set as seed regions (Figure 1). The Anatomical Automatic Labeling atlas was used to define the bilateral hippocampus. Seed-based analyses were performed. Mean time courses from all voxels within the unilateral hippocampus were extracted and used as reference time courses. The Pearson correlation coefficient was calculated between the mean time courses of each reference and voxel. Subsequently, Fisher's z transformation was applied to normalize the original correlation maps. The FC maps of the two regions were established and then analyzed in SPM12 using the analysis of variance model for calculating the difference between group-level functional maps. Brain regions were considered significant within a threshold of *p* < 0.05 after the false discovery rate was corrected for multiple comparisons and cluster size of >50.

### Statistical analysis

Data were represented as the mean ± standard deviation and analyzed by using SPSS version 20.0. Independent sample *t*-test and chi-square test were used to evaluate differences in demographic and clinical characteristics. The Mann–Whitney *U*-test was used to analyze the nonparametric data. A two-tailed Pearson's correlation analysis was used to obtain the correlations between the FC strength values of the significant brain regions and the cognitive scores. The significance level was set to 0.05.

## Results

### Demographic and clinical characteristics of the subjects

All the participants underwent MRI scanning, while one subject in each group was excluded due to excessive head motion during fMRI scanning. Mean interval between aneurysm rupture and MRI acquirement was 23.9 months. The clinical and demographic features of the remaining subjects are shown in Table 1. There was no significant difference in age, sex, and education level.

**Table 1:** Clinical features of all the participants.

	**Patients with ruptured ACoA aneurysm**	**Controls**	***P* value**
Age (year)	57.3 ± 9.8	53.3 ± 7.2	0.12
Gender (male/female)	15/11	10/9	0.77
Education (years)	9.1 ± 4.3	9.4 ± 4.1	0.623
Hunt-Hess on admission			
< 3	23	-	
3	2	-	
>3	1	-	
Size of aneurysm			
≤ 5 mm	12	-	
5–10 mm	13	-	
>10 mm	1	-	
Aneurysm treatment			
Coiling	8	-	
Clipping	18	-	
MoCA	23.88 ± 5.37	29.42 ± 0.84	< 0.01
SMCQ	4.16 ± 3.88	0.11 ± 0.32	< 0.001

The values were represented as mean ± standard deviation. ACoA, anterior communicating artery; MoCA, Montreal Cognitive Assessment; SMCQ, Subjective Memory Complaints Questionnaire.

### Comparisons of the intrinsic connectivity between the two groups

**Figure 2:**
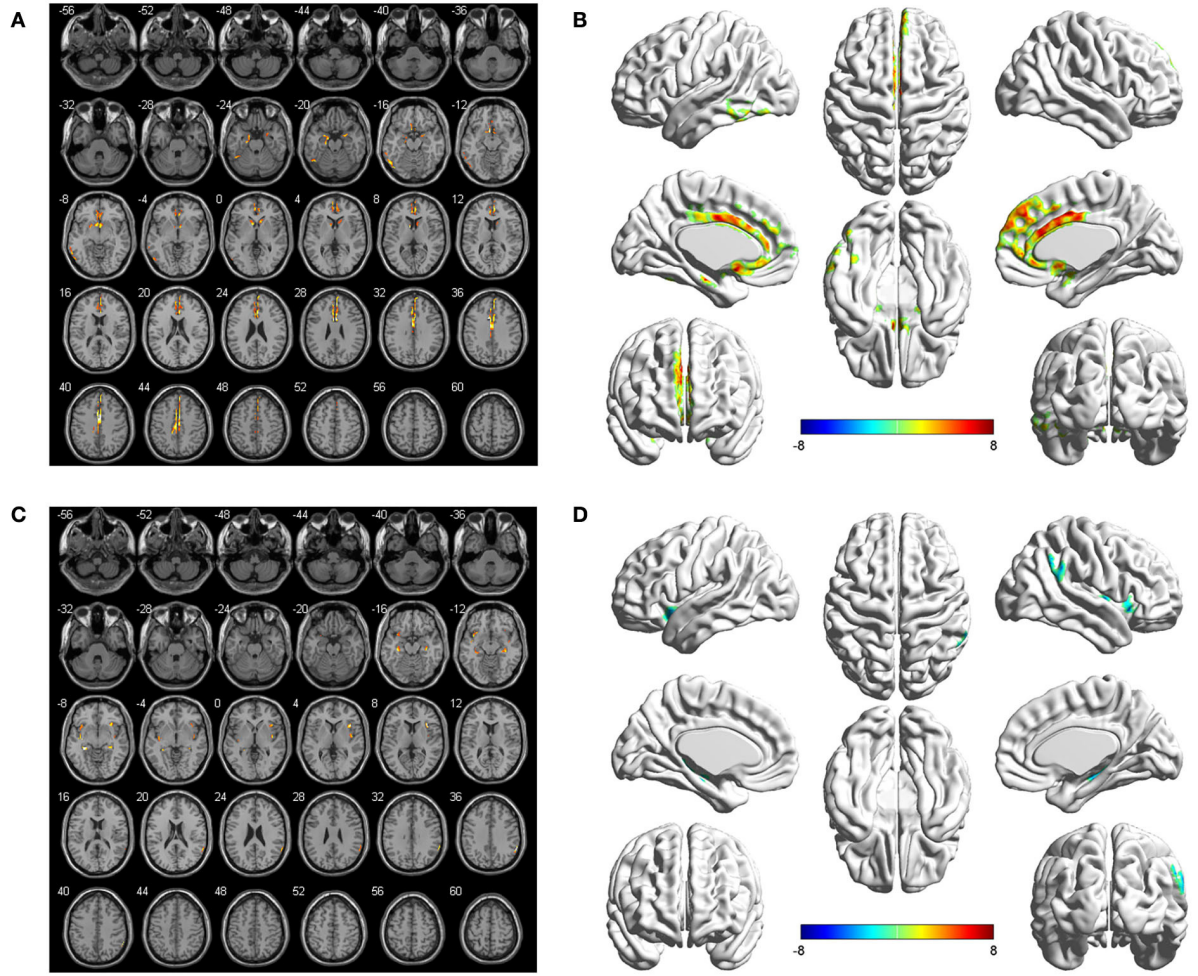
Differences in the left hippocampal connectivity between patients with ruptured ACoA aneurysm and healthy controls. Compared with the control group, brain regions labeling with color indicate decreased (upper panel) or increased functional connectivity (lower panel) in patients with ruptured ACoA aneurysm. Results were displayed in 2D **(A,C)** and 3D **(B,D)**, respectively. The displaying threshold was set to p < 0.05, false discovery rate corrected, and cluster size >50. Details of abnormal connectivity regions are shown in Table 2. ACoA, anterior communicating artery.

**Figure 3:**
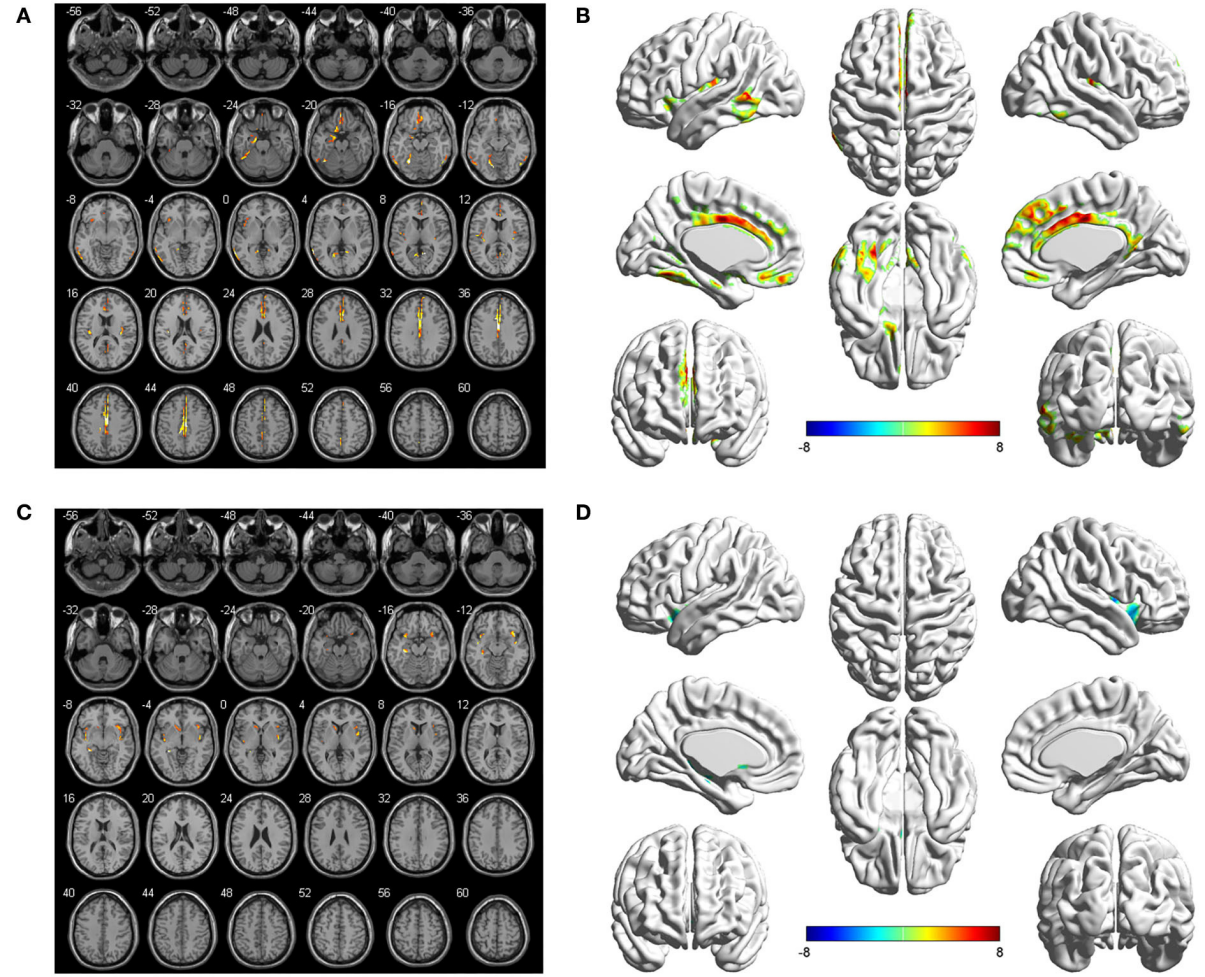
Differences in the right hippocampal connectivity between patients with ruptured ACoA aneurysm and healthy controls. Compared with the control group, brain regions labeling with color indicate decreased (upper panel) or increased functional connectivity (lower panel) in patients with ruptured ACoA aneurysm. Results were displayed in 2D **(A,C)** and 3D **(B,D)**, respectively. The displaying threshold was set to *p* < 0.05, false discovery rate corrected, and cluster size >50. Details of abnormal connectivity regions are shown in Table 2. ACoA, anterior communicating artery.

The seed-to-voxel FC analyses showed similar changes in the brain network of bilateral hippocampus. Specifically, when the left hippocampus was defined as ROI, reduction in intrinsic connectivity was found in bilateral anterior cingulate cortex (ACC), bilateral middle cingulate cortex (MCC), bilateral SFGmed, and left ITG in the group of ruptured ACoA aneurysm as compared to the healthy controls. Conversely, significantly increased functional brain connectivity was observed between the left hippocampus and the bilateral insula (Figure 2 and Table 2). Similar to the results of the left hippocampus-based FC analysis, decreased cerebral intrinsic connectivity in bilateral ACC, bilateral MCC, bilateral SFGmed, and left ITG was also uncovered with a seed placed in the right hippocampus. In addition, we revealed FC strength decline in the right hippocampus-left fusiform gyrus, and the right hippocampus-left middle temporal gyrus. Compared with healthy controls, hippocampal hyperconnectivity with bilateral insula and left hippocampus was found in patients with ruptured ACoA aneurysm (Figure 3 and Table 3).

**Table 2:** Brain regions showing significant differences of the left hippocampus-based functional connectivity in patients with ruptured ACoA as compared to the healthy controls.

**Brain region**	**Number of voxels**	**Peak MNI coordinates**			**Peak T value**
		**X**	**Y**	**Z**	
Middle and anterior cingulate cortex	689	−8	12	36	9.30
Medial surperior frontal gyrus	205	4	64	10	7.33
Left inferior temporal gyrus	51	−58	−44	−18	6.90
Right insula	84	38	18	6	−9.51
Left insula	53	−38	8	−12	−5.37

ACoA, anterior communicating artery; MNI, Montreal Neurological Institute.

**Table 3:** Brain regions showing significant differences of the right hippocampus-based functional connectivity in patients with ruptured ACoA as compared to the healthy controls.

**Brain region**	**Number of voxels**	**Peak MNI coordinates**			**Peak T value**
		**X**	**Y**	**Z**	
Left fusiform gyrus	96	−38	−58	−20	7.69
Left middle and inferior temporal gyrus	128	−64	−52	4	7.85
Middle and anterior cingulate cortex, medial surperior frontal gyrus	969	4	−16	46	11.11
Right insula	99	40	16	−12	−7.57
Left hippocampus	64	−32	−36	−8	−9.55
Left insula	93	−44	−2	−14	−7.10

ACoA, anterior communicating artery; MNI, Montreal Neurological Institute.

### Correlations between FC strength and cognitive function in the patients' group

**Figure 4:**
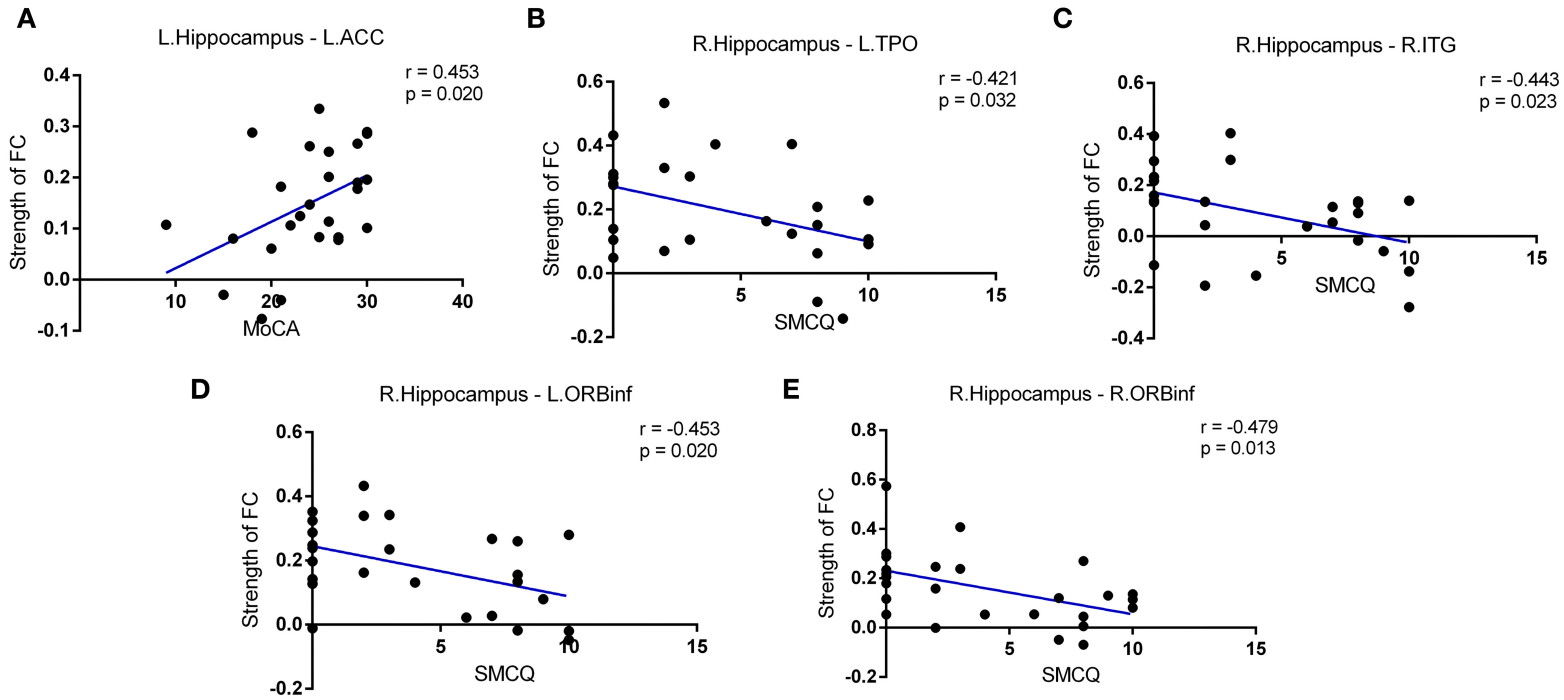
Correlations between intrinsic connectivity strength and cognitive function in patients after the rupture of ACoA aneurysm. **(A)** Positive correlation between MoCA scores and connection between left hippocampus-left ACC. Negative correlations between SMCQ scores and right hippocampus-left TPO **(B)**, right hippocampus-right ITG **(C)**, and right hippocampus-bilateral ORBinf **(D,E)** connectivity strength. ACoA, anterior communicating artery; MoCA, Montreal Cognitive Assessment; ACC, anterior cingulate cortex; SMCQ, Subjective Memory Complaints Questionnaire; ITG, inferior temporal gyrus; TPO, temporal pole; ORBinf, orbital part of inferior frontal gyrus.

We then performed correlation analyses between hippocampal connectivity and cognitive or memory performance in patients suffering from the rupture of ACoA aneurysm. As shown in Figure 4, a connection between the left hippocampus and the left ACC was found to be positively correlated with the MoCA total score. In addition, we also discovered that the strength of intrinsic connectivity between the right hippocampus and multiple brain regions was negatively correlated with SMCQ scores, including left temporal pole, right ITG, left orbital part of inferior frontal gyrus (ORBinf), and right ORBinf (r = −0.421, r = −0.443, r = −0.453, and r = −0.479, respectively).

## Discussion

To the best of our knowledge, this is the first study to explore the hippocampal connectivity with respect to patients with ruptured ACoA aneurysm. Consistent with our hypothesis, this study primarily uncovered significantly decreased FC in multiple brain regions, including bilateral ACC, bilateral MCC, bilateral SFGmed, and left ITG. Besides, the altered patterns of connectivity were largely overlapped in bilateral hippocampus. Conversely, the enhancement of connectivity strength between the hippocampus and the insula was found in the ACoA aneurysm group in comparison with healthy controls. Furthermore, hippocampal resting hypoconnectivity with several brain regions was significantly correlated with their memory loss.

Patients after the rupture of ACoA aneurysms are frequently reported to be associated with follow-up cognitive dysfunction, so the cognitive rehabilitation is necessary for them. Currently, functional brain network-directed neuromodulation has shown great promise in various cognitive disorders (Gimbel et al., 2021; Kolskår et al., 2021; Clancy et al., 2022). Several fMRI studies have shed light on this issue, and they revealed abnormalities of cognition-related networks in patients with aneurysmal SAH as compared to the control group. However, patients enrolled in these studies suffer from different locations of aneurysm that might cause diverse structural and functional brain damages. Meanwhile, some common factors contributing to cognitive declines such as hydrocephalus and epileptic seizure were also not ruled out. Therefore, to date, effective treatment options are still lacking due to the deficiency of knowledge about neural mechanisms. These confounding factors were well controlled in our recent resting-state fMRI study, and we provided evidence of aberrant SFGmed-based functional networks in patients with ruptured ACoA aneurysm. In a previous study, subjective memory complaints were determined in 44.4% of all patients. Hippocampus is one of the most recognized core regions responsible for memory function, and it is particularly susceptible to aneurysmal SAH insult. In addition, we noticed artifacts caused by surgical materials that were adjacent to the SFGmed. Given that, we extended to investigate the alterations of hippocampal connectivity in these patients following the rupture of ACoA aneurysm.

Consistent with our previous findings, intrinsic connectivity between hippocampus and SFGmed, a subdivision of mPFC, was also decreased in patients after the rupture of ACoA aneurysm in comparison with healthy controls. These two brain structures are known to regulate a variety of cognitive processes. Structural injury or functional hypoactivation of them has been linked to cognitive impairment underlying various neurologic diseases (Robin et al., 2015). Evidence from the FC analyses has also suggested that hippocampus–mPFC interactions are important to cognitive maps (Das and Menon, 2021; Zheng et al., 2021). Therefore, it is plausible that the neural activity of SFGmed and hippocampus decreases in synchrony in patients with ruptured ACoA.

In this study, we found that the patients with aneurysmal SAH showed significantly lower FC with the bilateral ACC and MCC no matter which side of the hippocampus was set as a seed (Figures 2, 3). As we know, the cingulate gyrus and hippocampus are important components of the limbic system and the hippocampal pathway to ACC participating in the formation of the Papez circuit in the human brain, which serves a principal function of memory retention and emotion control (Marchesi et al., 2022; Rolls et al., 2022). Evidence from animal studies suggests that hippocampal terminations innervated most of the excitatory neurons within ACC, suggesting strong excitatory effects on ACC neurons following hippocampal activity (Wang and Ikemoto, 2016; Wang et al., 2021). Predictably, if the hippocampus is compromised under pathological conditions, such as cerebral aneurysm rupture, the ACC is very likely to present hypoactivity. In agreement with this speculation, several diffusion tensor imaging studies of cerebral white matter changes in patients with SAH also provided robust structural evidence (Hong et al., 2012; Jang et al., 2014; Lee et al., 2020; Premat et al., 2021). For example, Jang et al. found injuries of the cingulum and fornix in patients after an ACoA aneurysm rupture, which were suggested to be associated with sustained memory impairment (Hong et al., 2012). In another research, the fractional anisotropy value and the volume of the mammillothalamic tract in the SAH group were significantly lower than that in the control subjects (Jang et al., 2014). Collectively, disruption of the Papez circuit in patients after the rupture of ACoA aneurysm may provide a crucial structural basis for hypoactivity between the hippocampus and cingulate gyrus.

Similar to the hippocampus, the main function of the insula is also related to intrinsic cognitive function. Consequently, low activity in the hippocampus and insula, and decreased connectivity between them, has been often linked to cognitive disorders. Conversely, the resting-state connectivity between each side of the hippocampus and bilateral insula was a significant increase in patients suffering from ruptured ACoA aneurysm in comparison with control subjects. Inconsistent findings of hypoconnectivity between the hippocampus and the insula in this study may be a compensatory mechanism for the abnormal Papez circuit caused by cognitive impairment. In addition, functional imaging studies have suggested that there exists abundant interinsular connectivity in the physiological state. This discovery partly explains the synchronized changes of the bilateral insular intrinsic connectivity.

There are several limitations in our study. First, extensive studies have provided evidence of differential connectivity patterns across the long axis of hippocampus, in which the anterior section of the hippocampus is associated with memory encoding and the posterior section of the hippocampus is associated with memory retrieval. In our study, although the left hippocampus and the right hippocampus have largely overlapped patterns of connectivity with other brain regions in patients with ACoA aneurysm in comparison with healthy controls, the anatomical and functional specialization of the hippocampus should be brought into focus. Second, memory loss is the main complaint of patients included in this study. Although we have used the SMCQ to assess the memory deficit and the MoCA scale to evaluate total cognitive function, scales for other cognitive domains preferably also need to be evaluated. Third, low connectivity of several brain regions within the Papez circuit was observed in patients after the rupture of ACoA aneurysm. However, undirected connection analysis exists inherent limitations. In the future, it is necessary to conduct further directed FC analysis that can better guide the neuromodulation treatment of cognitive impairment.

In summary, this study provides evidence of a decline in intrinsic connectivity between hippocampus and multiple brain regions in patients with a history of ruptured ACoA aneurysm. Most of the cortical hypoconnectivity is mainly located in the Papez circuit. Our findings implicate that decreased FC within the Papez circuit may be associated with cognitive impairment following the rupture of ACoA aneurysm, which we hope will contribute to future translational therapy options after aneurysmal SAH.

## Data availability statement

The original contributions presented in the study are included in the article/supplementary material, further inquiries can be directed to the corresponding authors.

## Ethics statement

The studies involving human participants were reviewed and approved by Local Ethics Committee of the First Affiliated Hospital of Fujian Medical University. The patients/participants provided their written informed consent to participate in this study.

## Author contributions

FC, DK, and DW conceived and designed the experiments. FC, LD, and JC drafted the manuscript and participated in data processing and statistical analysis. DK revised the manuscript. YK performed the MRI scanning. TY, LY, and YL assessed the cognitive function. FC, ZL, DK, TY, YK, YL, LY, DW, JC, and LD helped to collect data on patients and healthy controls. All authors read and approved the final manuscript.

## Funding

This study was supported by grants from the National Natural Science Foundation of China (81870930, 82171327, and 81901338), the Natural Science Foundation of Fujian Province (2021J01217), and the Technology Platform Construction Project of Fujian Province (2021Y2001).

## Conflict of interest

The authors declare that the research was conducted in the absence of any commercial or financial relationships that could be construed as a potential conflict of interest.

## Publisher's note

All claims expressed in this article are solely those of the authors and do not necessarily represent those of their affiliated organizations, or those of the publisher, the editors and the reviewers. Any product that may be evaluated in this article, or claim that may be made by its manufacturer, is not guaranteed or endorsed by the publisher.
